# T214. STIGMA ABOUT SCHIZOPHRENIA: THE EFFECTS OF DIAGNOSTIC LABELS, SYMPTOMS, AND ILLNESS PHASE

**DOI:** 10.1093/schbul/sby016.490

**Published:** 2018-04-01

**Authors:** Christopher Groot, Kelton Hardingham

**Affiliations:** 1 Melbourne School of Psychological Sciences, The University of Melbourne; 2 Melbourne School of Psychological Sciences

## Abstract

**Background:**

Schizophrenia is possibly the most stigmatised of all mental disorders. It has been argued that the label of schizophrenia itself has become stigmatically charged through over a century of association with misleading and sensationalist representations in news media and pop-culture. There has, therefore, been considerable recent debate on the topic of changing the label of schizophrenia. Label change advocates argue that public stigma about schizophrenia would be reduced should the label be replaced with either an eponymous or descriptive alternative. However, few empirical studies to date have directly investigated this possibility. Moreover, no known single study has investigated the effects of descriptive and eponymous relabelling of schizophrenia on public stigma as a function of symptomatology and illness phase. The current study aimed to fill this gap in the literature.

**Methods:**

Australian university students (m = 181, f = 176, other/unspecified = 4, Mage = 19.58 years, age range 17–60 years) participated in the study by reading a brief vignette depicting a protagonist with schizophrenia, and by subsequently completing a number of stigma measures. Participants were randomly allocated to vignette conditions that varied systematically by disorder label (schizophrenia vs eponymous label vs descriptive label), symptoms (positive vs negative symptoms), and illness phase (active vs remittent symptoms). Stigmatised thoughts, attitudes, and behaviour were measured using the Social Distance Scale (SDS; Link et al., 1987) and selected items forming six factors (Fear/Dangerousness, Help/Interaction, Responsibility, Forced Treatment, Pity, and Anger; Brown, 2008) from the Attributional Questionnaire (AQ; Corrigan et al., 2004). Socially desirable response bias and familiarity with mental illness were controlled using the Marlow-Crowne Social Desirability Scale (Crowne & Marlow, 1960), and Level-of-Contact Report (Holmes, Corrigan, Williams, Canar & Kubiak (1999).

**Results:**

Data was analysed with a series of ANCOVA and ANOVA analyses. No group differences in either SDS or AQ scores were observed as a function of disorder label. Positive symptoms were observed to elicit significantly greater levels of fear/dangerousness responses and endorsement of forced treatment when compared to negative symptoms. However, negative symptoms elicited significantly greater anger responses. While only these isolated differences on stigma measures were observed for symptom profile contrasts, vignettes depicting an active illness phase elicited significantly greater levels of stigma on the SDS and most AQ factors when compared with vignette conditions depicting a symptomatically remittent protagonist. Lastly, no statistically significant interactions were observed between labels, symptomatology and illness phase.

**Discussion:**

The results suggest that diagnostic label change may not be an effective strategy to reduce public stigma about schizophrenia in western countries. It is important to note, however, that public stigma elicitation is just one of the numerous considerations as regards the utility of label change, and that others, such as consumer perspectives, hold value independent of the current findings. The current findings also highlight that the symptomatology and illness phase of schizophrenia are likely to be implicated in eliciting public stigma about the disorder, and are worthy of further attention. While the current study investigated these phenomena at the broad symptom profile level, future research should investigate individual symptoms and their subtypes discretely, in order to inform a comprehensive, symptom-focussed model of elicited public stigma pertaining to schizophrenia.

